# Quantification of Mature MicroRNAs Using Pincer Probes and Real-Time PCR Amplification

**DOI:** 10.1371/journal.pone.0120160

**Published:** 2015-03-13

**Authors:** Tinghua Huang, Jun Yang, Guopin Liu, Wei Jin, Zhi Liu, Shuhong Zhao, Min Yao

**Affiliations:** 1 Black Pig Research Institute, Yangtze University, Jingzhou, Hubei, China; 2 College of Animal Science, Yangtze University, Jingzhou, Hubei, China; 3 Key Laboratory of Agricultural Animal Genetics, Breeding, and Reproduction of Ministry of Education, Huazhong Agricultural University, Wuhan, Hubei, China; Temasek Life Sciences Laboratory Ltd, SINGAPORE

## Abstract

The robust and reliable detection of small microRNAs (miRNAs) is important to understand the functional significance of miRNAs. Several methods can be used to quantify miRNAs. Selectively quantifying mature miRNAs among miRNA precursors, pri-miRNAs, and other miRNA-like sequences is challenging because of the short length of miRNAs. In this study, we developed a two-step miRNA quantification system based on pincer probe capture and real-time PCR amplification. The performance of the method was tested using synthetic mature miRNAs and clinical RNA samples. Results showed that the method demonstrated dynamic range of seven orders of magnitude and sensitivity of detection of hundreds of copies of miRNA molecules. The use of pincer probes allowed excellent discrimination of mature miRNAs from their precursors with five Cq (quantification cycle) values difference. The developed method also showed good discrimination of highly homologous family members with cross reaction less than 5%. The pincer probe-based approach is a potential alternative to currently used methods for mature miRNA quantification.

## Introduction

MicroRNAs (miRNAs) are endogenous, short RNA molecules (19 to 24 nucleotides) that serve as sequence-specific, post-transcriptional regulators of protein-coding genes. Identifying miRNA expression patterns is important in miRNA research. Northern blot was used as the “gold standard” method for miRNA quantification in early miRNA studies [1–3]. However, this approach is complicated and time consuming. In addition, the usage of this approach is restricted by its low sensitivity in quantifying low-abundant miRNAs. Liquid northern hybridization is a simplified method that uses fluorescently labeled oligonucleotide probes [4]. This method can complete the detection process within a few hours and simultaneously quantify multiple miRNAs in a single tube. Highly sensitive, polymerase amplification-based methods have been reported to complement hybridization-based miRNA detection [5–18]. In these methods, the miRNAs are usually first extended or used as templates to generate long sequences that can be amplified by polymerase. These methods include RNA tailing [5], stem—loop primer reverse transcription [6–11], adaptor ligation [12], tailed primer extension [13, 14, 18], padlock probe ligation [15], dual probe ligation [16, 17], and dumbbell-shaped DNA probe-directed rolling circle amplification [19]. Recently, Balcells et al. describe a miR-specific RT-qPCR technique which claimed as one of the best performing methods [20]. The method is as specific as stem-loop RT-PCR and the reverse transcription is performed with a universal primer and is therefore optimal for analysis of miRNAs for high-throughput screening. In addition, microfluidics and nanotechnology approaches [21], surface plasmon resonance biosensor [22] and the widely used next-generation sequencing technique [23] have also been proposed. These methods provide innovative and robust solutions for rapid and accurate miRNA quantification. In this study, we designed a novel, pincer probe-based real-time PCR amplification assay to quantify mature miRNAs. Test assays demonstrated that the pincer probe-based method can specifically quantify mature miRNAs from the miRNA precursors. The method can also discriminate miRNA homologs from the same miRNA family. The method provides a simple but robust way to quantify miRNAs.

## Results

### Design of the pincer probe-based miRNA detection system

**Fig 1:**
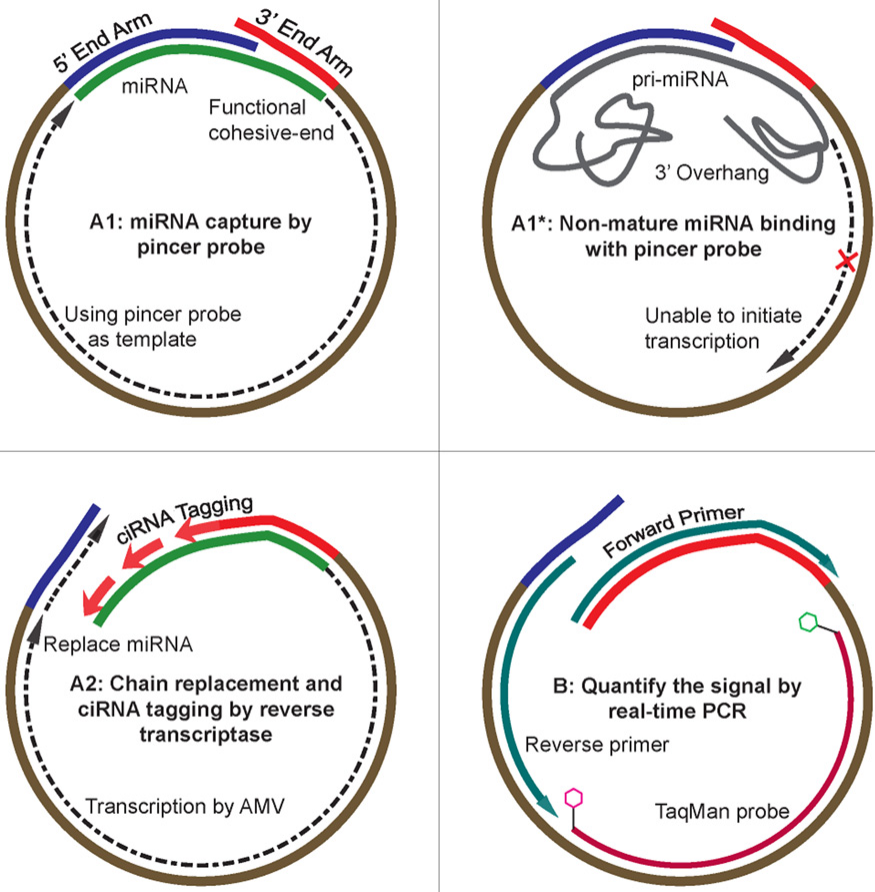
Schematic of the pincer probe-based miRNA quantification system. The pincer probe-based miRNA quantification assay included two steps: A) pincer probe reverse transcription and B) TaqMan real-time PCR. First, the pincer probe captured the target miRNA by undergoing a “pincer-like movement” and initiated transcription using the miRNA as a primer and the pincer probe as a template (A1). Although they can bind to the pincer probe, the nonmature miRNAs cannot initiate reverse transcription because of the 3′ end overhang (A1*). Then, the reverse transcriptase displaced the miRNA sequence complementary to the 5′ arm of the pincer probe that was encountered during synthesis and added a ciRNA tag to the 3′ end of the pincer probe (A2). Finally, the ciRNA-tagged pincer probes were quantified using conventional TaqMan qPCR assays that include the ciRNA-specific forward primer, the reverse primer, and dye-labeled TaqMan probes (B).

**Fig 2:**
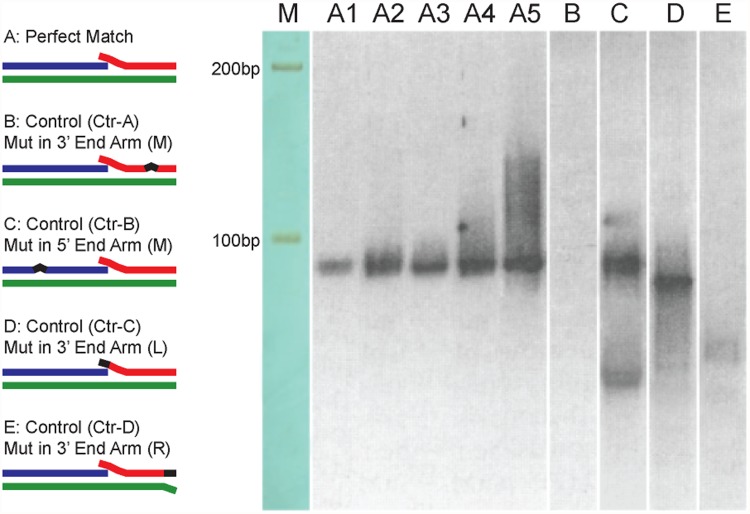
Testing the miRNA quantification system using pincer probes and controls. The lane designated “M” represents the size maker with an upper band of 200 bp and a lower band of 100 bp. The perfectly matched pincer probe and its target miRNA were actively reverse transcribed and were ciRNA tagged (lanes A1 to A5). The total amounts of miRNA in lanes A1 to A5 were 0.5, 1, 2, 4, and 8 pmol, respectively. The quantified amounts for bands A1 to A5 were 335.06, 662.25, 676.20, 1,345.30, and 2,547.50, respectively (Gel-Pro Analyzer). The mutations in the control probes were located in the middle of the arms (Ctr-A in the left arm and Ctr-B in the right arm) or in the 3′ cohesive end of the miRNA/pincer-probe duplex (Ctr-C in the pincer probe and Ctr-D in miRNA). Reverse transcription of miRNA with pincer-probe controls A, C and D (lanes B, D and E) did not produce correct products required for miRNA quantification. The pincer-probe control B (lane C) produced observable signal indicated that the assay may less to be precise if the mutation is close to the 5′ end. The reverse transcription product was detected using chemiluminescent photography.

A DNA pincer probe was designed to specifically recognize the target miRNA (Fig. 1A1 and S1 File). The probe contains two arms that are perfectly complementary to the target miRNA. The 5′ arm was longer while the 3′ end was significantly shorter. A primer binding site and a TaqMan probe binding site were also designed into the probe for real-time PCR amplification. Four pincer probes with separate mutations in each arm served as controls (Fig. 2, B to E; S1 File).

To quantify the miRNA, the two arms of the pincer probe were annealed to their targets by initially incubating at 40°C and then at 25°C. Reverse transcription was performed using the Avian Myeloblastosis Virus (AMV) reverse transcriptase to add ciRNA tags (sequence tags complement to the miRNA) to the pincer probe (Fig. 1A2). Long sequences that linearly reflect miRNA quantity were generated by ciRNA tagging. TaqMan real-time PCR amplification was then employed to quantify ciRNA-tagged pincer probes (Fig. 1B). A PCR was conducted to specifically amplify the ciRNA-tagged probes. A forward primer was used to bind to the ciRNA tags, and a reverse primer and a TaqMan probe were used to bind to the pre-designed sites in the pincer probe. The pincer probes without ciRNA tags cannot be amplified with this method. The PCR product exponentially accumulated at each round of amplification based on the quantity of ciRNA-tagged probes. This accumulation allowed miRNA quantification.

### Movement of the pincer probe

The two arms of the pincer probe were designed to have different melting temperatures. The longer 5′ arm annealed to the 5′ end portion of the target miRNA when incubated at 40°C, and then the shorter 3′ end arm of the pincer probe annealed to the 3′ end portion of the target miRNA when incubated at 25°C (The anneal temperature was predicted by the UNAFold software package, http://mfold.rna.albany.edu). There is one base pair overlap between the 3′ and 5′ end arms of the pincer probe. This design indicates that the last nucleotides of the 3′ end arm cannot bind to the target miRNA sequence because their binding sites were sequestered by the 5′ end arm of the pincer probe (Fig. 1A1). The pincer probe and the target miRNA formed a pincer-like RNA/DNA hybrid duplex (Fig. 1A1). We expected that the unpaired 3′ end arm of the pincer probe will block the initiation of reverse transcription. The elaborate miRNA/pincer probe duplex can be formed by controlling the cool-down rate with stepwise incubations at 42°C, 37°C, and then 25°C.

### Strand displacement and ciRNA tagging

The 3′ end of the miRNA base paired with the pincer probe and initiated transcription immediately after the formation of the miRNA/pincer probe duplex (Fig. 1A1). The AMV reverse transcriptase (New England Biolabs) exhibited strong strand displacement activity at temperatures between 20°C and 37°C. When the AMV reverse transcriptase encountered the 5′ end of the miRNA/pincer probe duplex, the AMV displaced the downstream miRNA sequence and synthesized a new chain using the pincer probes as templates (Fig. 1A2). This phenomenon allowed the release of the 5′ end of the miRNA that was sequestered by the 5′ arm of the pincer probe. The unpaired 3′ end (last nucleotides) of the pincer probe bound to the target sites and initiated transcription, which resulted in the addition of a new tag sequence to the 3′ end of the pincer probe (Fig. 1A2). Strand displacement and ciRNA tagging only occurred when a correct miRNA/pincer probe duplex was formed (Fig. 1A1). Although they can bind to the pincer probe, the non-mature miRNAs cannot initiate reverse transcription because of the 3′ end overhang (Fig. 1A1*). This design enables the specific addition of ciRNA tags to the pincer probes within the correctly assembled miRNA/pincer probe duplex.

### Validation of the reverse transcription product

A synthetic pseudo-miRNA was used to test whether or not the pincer probe system is functional (S1 File). Aside from the pincer probe that was designed to specifically bind the target miRNA, four synthetic pincer probe controls whose arms were introduced with artificial mutations were also used (Fig. 2, B to E; S1 File). The reverse transcription product of the pincer probe system using the synthetic pseudo-miRNA as a substrate was detected by digoxigenin blot. As predicted, chain replacement and ciRNA tagging occurred in the presence of the target miRNA in a quantitative fashion (Fig. 2, lanes A1 to A5). This result indicates the excellent ability of the pincer probe to capture the target miRNA. By contrast, no chain replacement and ciRNA tagging occurred in the pincer probe controls. This result indicates that these two phenomena are exclusive to the correctly assembled miRNA/pincer probe duplexes (Fig. 2, lanes B to E). In addition, the control-D (Fig. 2E) that simulated the miRNA-like sequence (miRNA precursors, pri-miRNAs and etc.) that bound to the pincer probe (Fig. 1A1*) showed no signal (Fig. 2, lane E). This result indicates that ciRNA tagging is exclusive to the mature miRNA sequences. No product of self-tagging caused by folding back of the 3′ end of the pincer probe was observed even at high concentrations of the pincer probe. The cross-tagging that used another pincer probe as a template yielded no product in the control reactions.

### Detection of miRNA in the absence or presence of total RNA

**Fig 3:**
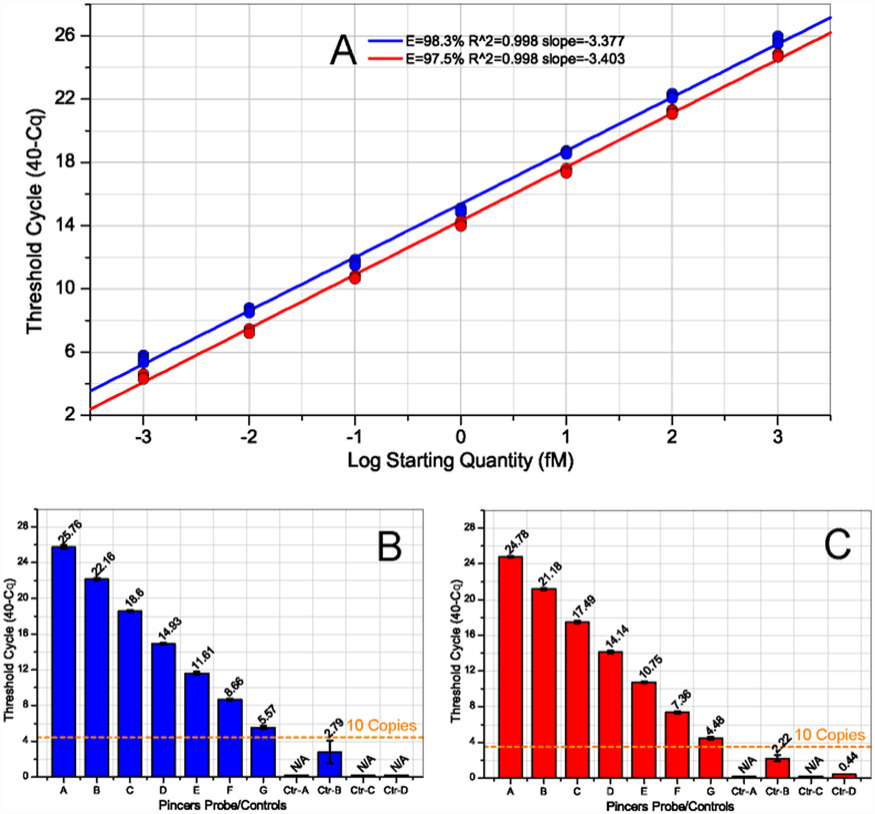
Dynamic range, specificity, and sensitivity of the pincer probe-based miRNA quantification assay. Plot A shows the correlation between the miRNA input and the Cq (threshold of cycle) values in the test assays which in the absence or presence of total RNA (Spike-In test). Plots B and C show the Cq values from the test assays using perfectly matched pincer probes or control pincer probes. The synthetic miRNA input ranged from 1.0 X 10^-3^ fM (about 60 copies per reaction) to 1.0 X 10^3^ fM in the PCR (lane A to G, perfectly matched pincer probes). The amount of synthetic miRNA for the four pincer-probe control test assays (Ctr-A to Ctr-D) was 10 pmol. The total RNA input for the Spike-In test assay was 1 μg. The amplification chart and raw data are available in S1 File.

The pincer probe system was tested with total RNA excluded from the reaction in vitro. The synthetic pseudo-miRNA did not contain any sequence from the transcriptome and can be directly used in the test. The synthetic pseudo-miRNA was diluted over seven orders of magnitude based on the quantity determined by the A260 absorbance and then measured as described above. The concentration of the synthetic pseudo-miRNA input ranges from 1.0 X 10^-3^ fM (about 60 copies per reaction) to 1.0 X 10^3^ fM. The results of the TaqMan assays showed excellent linearity between the log-transformed concentrations of the input miRNA and the qPCR Cq (quantification cycle) values (E = 98.3%, R^2^ = 0.998, Fig. 3A and S1 File), indicating that the dynamic range of the assay was over seven orders of magnitude. Each reaction was independently replicated four times. The standard deviations of the Cq values were all below 0.5 (S1 File). The no-template controls showed no detectable signals in any of the samples, confirming the high specificity of the assay for primed RNA. The results of the TaqMan assays using the synthetic pseudo-miRNA with pincer-probe controls (Fig. 3B, Ctr-B) revealed observable signals (synthetic miRNA input was 10 pmol). However, the accumulated intensity of fluorescence was just above 100 with Cq values higher than 35. This value is significantly lower than the perfectly complementary pincer probe. These results indicate that the ciRNA tagging of the pincer probe controls was nearly undetectable (Fig. 3B and S1 File).

A Spike-In test was performed, in which the reactions were performed in the presence of total RNA to simulate a typical reverse transcription reaction. Unlike the endogenous, short, RNA molecules, the synthetic pseudo-miRNA was supposed to be absent in the total RNA prepared from tissues. To conduct the Spike-In test, the synthetic pseudo-miRNA standard was serially diluted over a range of seven orders of magnitude. The standard was mixed with an equal amount of total RNA prepared from tissues and measured using the established approach. The concentration of the synthetic pseudo-miRNA input ranges from 1.0 X 10^-3^ fM (about 60 copies per reaction) to 1.0 X 10^3^ fM. The results of the TaqMan assays showed excellent linearity between the log-transformed concentrations of the input miRNA and the Cq values (E = 97.5%, R^2^ = 0.998, Fig. 3A and S1 File). Compared with the total RNA absent test, the standard deviation of the Spike-In test was almost the same among the four independent replicates, even for the low-concentration miRNA standard (average 0.14 versus 0.12, S1 File). The no-template controls did not amplify in the Spike-In test. The pincer probe control (Fig. 3C, Ctr-B) that contained a mutation in the middle of the longer arm (5′ end arm) showed an exponential amplification. However, the accumulated fluorescence intensity was still lower than the lowest-concentration miRNA standard (Fig. 3C and S1 File). We concluded that the dynamic range of the Spike-In test was over seven orders of magnitude. This assay was sensitive enough to accurately detect as few as hundreds of copies of miRNA molecules.

### Performance of the pincer probe with real-world miRNAs

**Fig 4:**
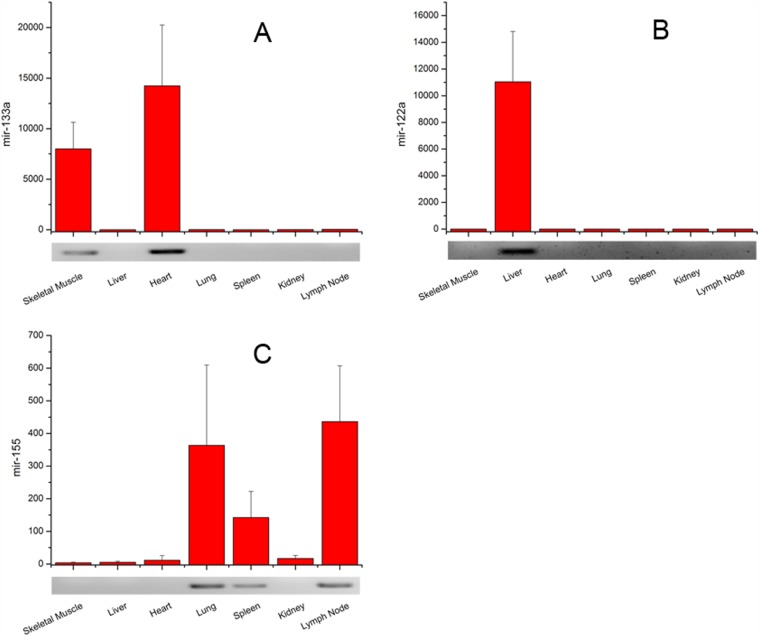
Quantification of miR-133a, miR-122a, and miR-155 expression in different tissues. A bar graph shows the normalized expression value detected by qPCR for each miRNA, and an autoradiogram shows the average signal of Northern blot for three repeated experiments. In the bar graph, the signal from nine tissues was normalized to the internal control of Met-tRNA. The background signal obtained from the sample containing H_2_O instead of RNA was set to zero.

We tested the capability of the pincer probe to detect changes in miRNA expression between different tissue samples. We constructed a pincer probe against miR-133a, which is specifically expressed in skeletal muscle tissues [24]. A clear miR-133a specific signal was observed in porcine skeletal muscle and heart tissues when 100 ng of total RNA was used. By contrast, no or a faint signal was observed in other tissues. The result agrees with the results of Northern blot analysis (Fig. 4A). Similarly, pincer probes directed against miR-122 and miR-155 were tested using 100 ng of total RNA isolated from seven porcine tissue samples. Results indicated that the miR-122 was specifically expressed in liver tissues while the miR-155 was specifically expressed in lymph node, lung, and spleen tissues. The results obtained using the pincer probe assays closely correspond to the results obtained from the Northern blots (Fig. 4B and C).

### Multiplex detection of the pincer probe method

Multiplex detection of miRNAs from total RNA can dramatically increase the throughput of the assay. The multiplex capability of the pincer probe real-time PCR assay was tested using pooled reverse transcription primers (the pincer probes). Nine miRNA assays were validated using porcine total RNA isolated from liver, heart and lung tissues. The results demonstrated that the selected miRNAs were abundantly expressed in these tissues with averaging Cq value of 26.4. The level of expression ranged from 21.8 Cq to 34.4 Cq. The miR-16 was the most abundant miRNA and let-7e was the least abundant miRNAs across all tissues. The overall level of miRNA expression was highest in lung and lowest in liver. Finally, the dynamic range of miRNA expression varied greatly from less than 3-fold (miR-20) to more than 25-fold (miR-21) among these tissues (Table 1). The performance of the multiplex tests was compared with the assays using each of the single reaction in the same sample. The results indicated that there was no significant difference between the Cq readout of the singleplex and multiplex assays (S1 File).

**Table 1:** Multiplex detection of miRNAs using pincer probe or stem-loop real-time RT-PCR.

MiRNA target	Pincer probe RT-PCR			Stem-loop RT-PCR		
	Liver*	Heart	Lung*	Liver	Heart	Lung
miR-16	24.3	21.8	23.7	24.0	22.2	22.6
miR-20	25.7	24.2	24.2	24.2	23.8	24.2
miR-21	24.8	26.6	21.9	23.9	24.9	22.1
miR-22	28.5	26.0	29.7	27.9	26.2	28.6
Let-7a	27.2	24.6	24.8	25.2	23.6	24.6
Let-7b	27.8	27.9	25.6	25.8	26.8	24.7
Let-7c	25.2	22.9	23.4	24.3	23.8	22.8
Let-7d	29.8	27.2	29.1	29.3	26.7	28.4
Let-7e	34.4	32.5	30.1	32.4	31.1	29.0

*Paired T-test was performed to show the difference between the pincer probe method and the stem-loop RT-PCR method. The signal of the pincer probe method is significantly lower than the stem-loop RT-PCR for the liver tissue (p-value < 0.01) and lung tissue (p-value < 0.01).

The stem-loop RT-PCR method developed by Chen et al. [8] was used as control method for quantification. Paired T-test was performed to show the difference between the pincer probe and the stem-loop miRNA quantification method. The signal of the pincer probe method was significantly lower than the stem-loop method for the liver tissue (1.19 Cq, p-value < 0.01) and the lung tissue (0.61 Cq, p-value < 0.01). No significant difference was observed in the detection of miRNAs from the total RNA isolated from heart tissue. The overall Pearson correlation efficiency between pincer probe based method and stem-loop based method was 0.98 indicate good agreement of the two methods. However, it is worthy to note that the one cycle difference of the averaged expression level between the two methods indicated that the quantification of miRNAs was compromised with the RT reaction using pincer probes.

### Specific detection of mature against precursor miRNAs

The capability of the pincer probe real-time PCR assay to discriminate mature against precursor miRNAs was investigated using 11 mature miRNAs and their corresponding precursors (miR-133a, miR-122, miR-155, and eight let-7 miRNAs). The same amount (approximately 6 × 10^3^ copies per reaction) of mature miRNAs and pre-miRNAs was individually quantified using pincer probe real-time PCR. Depending on the 11 pre-miRNAs examined, the signal was almost under detectable (10 out of 11 are higher than 35 cycles). The mature miRNAs were detected 5.7 to 10.1 cycles (average deta Cq of 8.8 cycles) earlier than the pre-miRNAs using pincer probe real-time PCR (Table 2).

**Table 2:** Discrimination between the mature and the precursor miRNAs using pincer probe or linear real-time RT-PCR.

MiRNA target	Pincer probe RT-PCR			Linear RT-PCR		
	Precursor*	Mature*	Delta Cq	Precursor	Mature	Delta Cq
mir-133	36.1	28.0	8.1	34.1	30.0	4.1
mir-122	38.5	29.1	9.4	36.5	33.4	3.1
mir-155	34.2	28.5	5.7	33.2	29.2	4.0
Let-7a	37.7	28.7	9.0	35.2	36.5	-1.3
Let-7b	39.4	29.3	10.1	37.5	32.5	5.0
Let-7c	40.0	30.2	9.8	37.0	35.0	2.0
Let-7d	35.5	28.1	7.4	33.0	34.0	-1.0
Let-7e	37.5	28.0	9.5	34.5	30.2	4.3
Let-7f	39.5	30.1	9.4	37.5	30.1	7.4
Let-7g	37.0	28.5	8.5	34.2	29.0	5.2
Let-7i	38.2	28.8	9.4	34.0	32.6	1.4

*Paired T-test was performed to show the difference between the pincer probe method and the linear RT-PCR method. The signal of the pincer probe method is significantly lower than the linear RT-PCR for the mature miRNAs (p-value < 0.01) and significant higher for the precursors (p-value < 0.01).

For comparison, we also evaluated the real-time PCR method using linear RT oligonucleotides (data shown in Table 2). This method use tailed linear primer for the reverse transcription and tailed linear primer for the real-time PCR. The result demonstrated that the signal of the pincer probe method is significantly lower than the linear RT-PCR for the mature miRNAs (p-value < 0.01) and significant higher for the precursors (p-value < 0.01). Furthermore, the assays using linear RT oligonucleotides were less discriminative (average delta Cq of 3.1 cycles) and failed to discriminate some of the mature miRNAs from their precursors, such as let-7a and let-7d (Table 2).

### Discrimination of let-7 miRNA homologs

The let-7 miRNA family consists of highly homologous miRNAs that differ by only a few nucleotides. The eight let-7 family miRNAs share up to 63.6% overall sequence identity, among which let-7a and let-7c, let-7a and let-7f, and let-7b and let-7f differ only by a single nucleotide. In this study, pincer probe real-time PCR assays for each let-7 miRNA were designed. Each assay was used to amplify all eight synthetic let-7 miRNAs (approximately 6 × 10^3^ copies of let-7 miRNA per reaction), and the detected signal was compared in Table 3. Most of the let-7 assays showed excellent discrimination against the homologous miRNAs with less than 5% nonspecific signal. The let-7c assay showed 5.07% nonspecific signal against let-7a. For let-7 miRNAs that differed by two nucleotides or more, these assays specifically detected the target miRNA with less than 1.00% cross target detection. This result suggests that the pincer probe real-time PCR assay can discriminate highly homologous miRNAs.

**Table 3:** Cross reaction (%) of each let-7 miRNAs by specific pincer probe real-time PCR assays.

Detection (%)	Let-7a	Let-7b	Let-7c	Let-7d	Let-7e	Let-7f	Let-7g	Let-7i
Let-7a	100	0.00	1.36	0.00	3.03	2.18	0.00	0.00
Let-7b	0.00	100	0.00	0.00	0.00	0.00	0.00	0.00
Let-7c	5.07	0.00	100	0.00	0.10	0.00	0.00	0.00
Let-7d	0.00	0.00	0.00	100	0.00	0.00	0.00	0.11
Let-7e	2.89	0.00	0.30	0.00	100	2.12	0.00	0.00
Let-7f	3.22	0.00	0.00	0.00	1.82	100	0.00	0.00
Let-7g	0.00	0.00	0.00	0.00	0.00	0.00	100	0.00
Let-7i	0.00	0.00	0.00	0.20	0.00	0.00	0.00	100

## Discussion

Determining miRNA expression profiles with high sensitivity and specificity has technical challenges. First, mature miRNAs are short, only 18–23 nucleotides. Second, the target sequence is also present in pri-miRNAs and pre-miRNAs. Third, miRNAs within the same family may differ by only one nucleotide (such as the Let-7 family). These challenges hinder the development of efficient miRNA quantification techniques. Given its high sensitivity, accuracy, and practical ease, real-time PCR is usually used for expression analysis in miRNA research [25]. The pincer probe real-time PCR assays presented in this study exhibited simplicity, excellent performance, and flexibility for designing any miRNAs. Using synthetic miRNAs, the pincer probe real-time PCR assay showed a wide dynamic range of at least seven orders of magnitude, high sensitivity of as few as hundreds of molecules per reaction, and amplification efficiency of higher than 95%. Twelve pincer probe real-time PCR assays were developed and tested using RNA samples extracted from porcine tissues. The expression level examined by the pincer probe real-time assays showed the same expression patterns as the methods quantified with Northern blot assay or other similar miRNA quantification approaches. These results indicated that the developed miRNA quantification method is valid.

The detection throughput was a major concern in the quantitative analysis of miRNA profiling. Parallel reverse transcription for a large number of miRNAs contained in a single sample has been reported recently. Thang and collaborators used a mixture of 220 individual stem-loop primers to multiplex the reverse transcription step [26, 27]. In the present study, nine miRNAs was detected simultaneously in the reverse transcription step and then quantified by the real-time PCR. We believed that once incorporated with the multiplexed detection system, our assay also held the possibility of being an assay of quantitative analysis of multiple miRNA molecules simultaneously.

Two steps in miRNA biogenesis produce sequences that are relevant to miRNA quantification. The first step is the cropping of the primary sequence that is transcribed from the miRNA locus in the genome. This step generates 70 nucleotides pre-miRNAs. The second step is the cleavage of the pre-miRNAs in the cytoplasm. This step yields 18 to 23 nucleotides mature miRNAs [28]. MiRNA biogenesis can be regulated by multiple mechanisms, resulting in nonlinear processing speed between each step and intermediary miRNA product accumulation in the middle of the pipeline [28]. Although the expression levels of precursor miRNAs often correlate with mature miRNAs [29, 30], miRNA maturation can be regulated in a way that precursor miRNAs are expressed but their mature forms are undetectable [11, 31]. The primary miRNA transcripts, the pri-miRNAs, and the complementary chain of the miRNA genomic locus are embedded with a short sequence piece that is exactly the same as the mature miRNA because of the linearity of miRNA biogenesis. These miRNA-like sequences are always present in variable amounts in the total RNA extracted from tissues. However, only the mature miRNA is functional and is of most interest. This condition presents a challenge to miRNA quantification techniques because the assay must be capable of exclusively detecting 18 to 23 nucleotides mature forms rather than the primary transcripts, pri-miRNAs or other miRNA-like sequences.

The present pincer probe system was designed to selectively quantify mature miRNAs. Through the pincer movement within the reaction, only the mature miRNAs can correctly form a miRNA/pincer probe duplex with a functional, 3′, cohesive-end site that can initiate transcription (Fig. 1A1 and A1*). Other miRNA-like sequences cannot initiate transcription because of the 3′ end overhang (Fig. 1A1*). As a result, these molecules cannot be detected by the assay. The capability of the pincer probe real-time PCR assay to discriminate mature against precursor miRNAs was also tested using synthetic mature miRNAs and their precursors. As expected, the pincer probe real-time assays were highly specific for the detection of mature miRNAs and not pri-miRNAs. This result indicates that the signal of the mature miRNAs detected by the pincer probe real-time assay is higher than that of the miRNA precursors (average 100 times higher). The capability of the assay to discriminate mature miRNAs and miRNA precursors is sufficient to meet the miRNA quantification demand in most experiments.

However, simultaneously discriminating a group of highly homogenous miRNAs (such as the let-7 family) remains challenging. MiRNAs are grouped into families based on an identical seed sequence that spans the 2 to 7 nucleotides at the 5′ end of the miRNAs. This sequence is a critical determinant of target recognition [32]. Although miRNAs from the same family are likely to share some common targets and functions, the expression of specific members of the family may be under specific regulation control and thus involved in different biological processes and disease states. Specific detection and discrimination of these miRNAs are required for research on miRNA functions and miRNA biogenesis. Previously, discrimination of let-7 homologs was achieved with TaqMan real-time PCR assays. After a miRNA-specific reverse transcription, a common reverse primer is used for PCR and a miRNA-specific TaqMan probe is required for discrimination [8]. We hypothesized and showed that the specificity of the assays can be achieved by using a miRNA-specific pincer probe. The pincer probe-based real-time PCR amplification assay can be used to specifically detect and discriminate all the let-7 miRNAs. The discriminating capability of the pincer probe miRNA quantification method is superior to that of the method reported by Wang et al., comparable with that of the methods developed by Chen et al. and Yao et al., and slightly lower than that of the method reported by Schmittgen et al. and Wan et al. [8, 10, 11, 13, 33]. In general, the pincer probe real-time PCR method can specifically detect and discriminate most miRNAs. Thus, this method is suitable for research on miRNA functions and miRNA biogenesis. A single nucleotide difference at the middle position can be easily distinguished by the pincer probe. However, a terminal mismatch, particularly at the 5′ end of the miRNA, would lead to poor discrimination.

## Materials and Methods

The DNA pincer probes and the miRNA standard were synthesized and quantified by Integrated DNA Technologies (S1 File). AMV reverse transcriptase was purchased from New England Biolabs (Catalog number: M0277). The Supermixes for real-time PCR were obtained from Bio-Rad Corporation (Catalog number: 170–8860). Porcine tissues were obtained from three matured healthy castrated crossbred pigs (Duroc × Large white × Landrace, three month old). All experiments complied with the policy on the care and use of laboratory animals and were approved by the Ethics Committee of Yangtze University and Huazhong Agricultural University. The animals were housed in floored indoor pens and were allowed free access to standard food and water at least a week before the experiment. The animals were euthanized by intravenous injection of pentobarbital sodium (150 mg kg^-1^ body weight). Within 5 min after death, the tissue samples were excised. The tissues were immersed in RNALater for 12 h at 4°C and then stored at −80°C until use. Total RNA from porcine tissue samples was isolated with Trizol reagent following the manufacturer’s protocol (Invitrogen) without any additional small RNA purification or enrichment process.

Reverse transcription was performed in a total reaction volume of 20 μL as follows. Briefly, 2 μL of RNA (100 ng), 2 μL of pincer probes (10 μM), and 4 μL of dNTP mix (2.5 mM each) were mixed into a nuclease-free microfuge tube. The mixture was added with nuclease-free H_2_O to final volume of 16 μL and then incubated under the following conditions: 5 min at 70°C, 2 min at 42°C, and 2 min at 37°C. The mixture was added with 2 μL of 10 X AMV reverse transcriptase reaction buffer, 1 μL of RNase inhibitor (10 units/μL), and 1 μL of AMV reverse transcriptase. A 20 μL aliquot of the reaction mixture was incubated for 30 cycles of 25°C for 30 s and 37°C for 2 min. Then, the enzyme was inactivated at 80°C for 5 min. No-template control reactions were included in all reverse transcriptase reactions. Reverse transcription with chemiluminescent photography was performed using almost the same reaction conditions, except the regular dNTP mix was replaced with a dNTP mix containing alkali-labile Digoxigenin-11-dUTP.

The products of reverse transcription were subjected to real-time PCR analysis. A 25μL aliquot of real-time PCR reaction mixture contained 12.5 μL of Bio-Rad iQ Supermix, 2 μL of reverse transcription product, 0.35 μL each of the reverse and forward primers (10 μM), 0.25 μL of the TaqMan probe (10 μM), and nuclease-free H_2_O. Cycling and quantification were performed on the BIO-RAD iQ5 Real-Time PCR system under the following conditions: 3 min at 95°C, followed by 40 cycles of 30 s at 95°C, 30 s at 60°C, and 45 s at 72°C. The real-time PCR product was checked by 3% agarose gel electrophoresis for specificity.

For the chemiluminescent photography assays, the reverse transcription products were separated by 15% polyacrylamide gel electrophoresis and then transferred to Hybond N^+^ membranes (Amersham) using a semidry blotter. The DIG-labeled probes were immunodetected with anti-digoxigenin-AP and then incubated with the chemiluminescence substrate CSPD. The membranes were exposed to X-ray films for 15 min to 25 min.

## Supporting Information

S1 File Supplemental material. (DOC)
