# Supplementary material for: Quantification of Mature MicroRNAs Using Pincer Probes and Real-Time PCR Amplification
Source: PLoS One. 2015 Mar 13;10(3):e0120160. doi: 10.1371/journal.pone.0120160 (PMC4359002; doi:10.1371/journal.pone.0120160)
Supplement: S1 File — (DOC) [file pone.0120160.s001.doc]

**Supplemental Material**

1. **Sequences of primer probes of the pseudo miRNA**

The randomly selected, synthetic, pseudo miRNA is from the intergenic region of a protein-coding gene and has been proven to be absent in total RNA.

Pseudo-miRNA: 5’ CAAGGAGAACUCAAGGUCAGCCCU 3’

Pincer Probe: CTTGAGTTCTCCTTGCCACGACCGAAATCCGACTTAGCCACATCGCTCAGACACCAAGGAGGGCTGACC

Forward Primer: 5’ CAAGGAGAACTCAAGGTCAGCC 3’

Reverse Primer: 5’ TTGCCACGACCGAAATCC 3’

TaqMan Probe: 5’ FAM-AGCCACATCGCTCAGACACCAAGG-TAMARA 3’

Control A: CTTGAGTTCTCCTTGCCACGACCGAAATCCGACTTAGCCACATCGCTCAGACACCAAGGAGGG*T*TGACC

Control B: CTTGA*C*TTCTCCTTGCCACGACCGAAATCCGACTTAGCCACATCGCTCAGACACCAAGGAGGGCTGACC

Control C: CTTGAGTTCTCCTTGCCACGACCGAAATCCGACTTAGCCACATCGCTCAGACACCAAGGAGGGCTGA*AG*

Control D: CTTGAGTTCTCCTTGCCACGACCGAAATCCGACTTAGCCACATCGCTCAGACACCAAGG*TC*GGCTGACC

1. **Test without total RNA, amplification chart and Cq values**


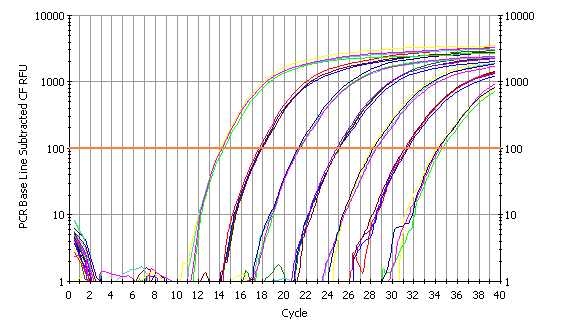


| Sample | Rep_1 | Rep_2 | Rep_3 | Rep_4 | NTC | Stdev |
| --- | --- | --- | --- | --- | --- | --- |
| A | 14.03 | 14.22 | 14.22 | 14.50 | N/A | 0.19 |
| B | 17.66 | 17.84 | 17.94 | 17.94 | N/A | 0.13 |
| C | 21.28 | 21.38 | 21.47 | 21.47 | N/A | 0.09 |
| D | 24.91 | 25.09 | 25.19 | 25.09 | N/A | 0.12 |
| E | 28.16 | 28.35 | 28.53 | 28.53 | N/A | 0.18 |
| F | 31.23 | 31.23 | 31.41 | 31.51 | N/A | 0.14 |
| G | 34.20 | 34.39 | 34.48 | 34.67 | N/A | 0.19 |
| Ctr-A | N/A | N/A | N/A | N/A | N/A | N/A |
| Ctr-B | 36.18 | 36.82 | N/A | 38.64 | N/A | 1.27 |
| Ctr-C | N/A | N/A | N/A | N/A | N/A | N/A |
| Ctr-D | N/A | N/A | N/A | N/A | N/A | N/A |

1. **Test with total RNA, amplification chart and Cq values**


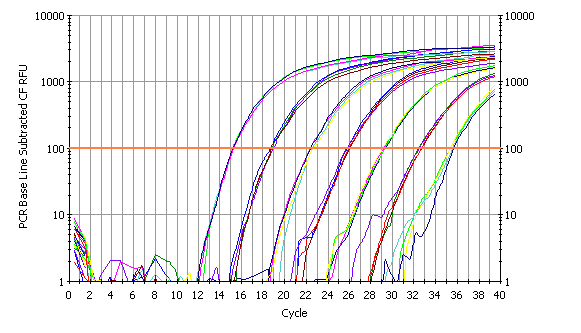


| Sample | Rep_1 | Rep_2 | Rep_3 | Rep_4 | NTC | Stdev |
| --- | --- | --- | --- | --- | --- | --- |
| A | 15.15 | 15.15 | 15.24 | 15.33 | N/A | 0.09 |
| B | 18.68 | 18.77 | 18.87 | 18.96 | N/A | 0.12 |
| C | 22.40 | 22.40 | 22.58 | 22.68 | N/A | 0.14 |
| D | 25.74 | 25.74 | 25.93 | 26.02 | N/A | 0.14 |
| E | 29.18 | 29.18 | 29.28 | 29.37 | N/A | 0.09 |
| F | 32.53 | 32.53 | 32.71 | 32.81 | N/A | 0.14 |
| G | 35.41 | 35.41 | 35.59 | 35.69 | N/A | 0.14 |
| Ctr-A | N/A | N/A | N/A | N/A | N/A | N/A |
| Ctr-B | 37.55 | 38.00 | N/A | N/A | N/A | 0.32 |
| Ctr-C | N/A | N/A | N/A | N/A | N/A | N/A |
| Ctr-D | N/A | N/A | 39.56 | N/A | N/A | N/A |

1. **Sequences of primer probes of the real-world miRNAs**

miR-133a-3p：5’ uuugguccccuucaaccagcug 3’

miR-133-FP: TTTGGTCCCCTTCAACCAGCT

miR-133-RP: AAAAGACACCAACACATCGCA

Probe: 5’ FAM-CAGGAGCCAGCCGTCGCACACCGT-TAMARA 3’

miR-133-PP: tgaaggggaccaaaAGACACCAACACATCGCAATCAGGAGCCAGCCGTCGCACACCGTcagctggtt

miR-122-5p: 5’ uggagugugacaaugguguuug 3’

miR-122-FP: TGGAGTGTGACAATGGTGTTTG

miR-122-RP: CTCCACCACGACCGAAATCCG

Probe: 5’ FAM-CAGGAGCCAGCCGTCGCACACCGT-TAMARA 3’

miR-122-PP: attgtcacactccaCCACGACCGAAATCCGACCAGGAGCCAGCCGTCGCACACCGTcaaacacca

miR-155-5p: 5’ uuaaugcuaaucgugauaggggu 3’

miR-155-FP: TTAATGCTAATCGTGATAGGGGT

miR-155-RP: TAAAGACACCAACACATCGCA

Probe: 5’ FAM-CAGGAGCCAGCCGTCGCACACCGT-TAMARA 3’

miR-155-PP: cacgattagcattaaAGACACCAACACATCGCAATCAGGAGCCAGCCGTCGCACACCGTacccctatc

let-7a-5p：ugagguaguagguuguauaguu

let-7a-PP: aacctactacctcaCCACGACCGAAATCCGACTTAGCCACATCGCTCAGACACCAAGGaactataca

let-7a-FP: TGAGGTAGTAGGTTGTATAGTTCC

let-7a-RP: TCACCACGACCGAAATCC

TaqMan Probe: 5’ FAM-AGCCACATCGCTCAGACACCAAGG-TAMARA 3’

let-7b-5p：ugagguaguagguugugugguu

let-7b-PP: aacctactacctcaAGACACCAACACATCGCAATCAGGAGCCAGCCGTCGCACACCGTaaccacaca

let-7b-FP: TGAGGTAGTAGGTTGTGTGGT

let-7b-RP: TCAAGACACCAACACATCGCA

TaqMan Probe: 5’ FAM-CAGGAGCCAGCCGTCGCACACCGT-TAMARA 3’

let-7c-5p：ugagguaguagguuguaugguu

let-7c-PP: aacctactacctcaAGACACCAACACATCGCAATCAGGAGCCAGCCGTCGCACACCGTaaccataca

let-7c-FP: TGAGGTAGTAGGTTGTATGGTTAC

let-7c-RP: TCAAGACACCAACACATCGCA

TaqMan Probe: 5’ FAM-CAGGAGCCAGCCGTCGCACACCGT-TAMARA 3’

let-7f-5p：ugagguaguagauuguauaguu

let-7f-PP: aatctactacctcaAGACACCAACACATCGCAATCAGGAGCCAGCCGTCGCACACCGTaactataca

let-7f-FP: TGAGGTAGTAGATTGTATAGTTAC

let-7f-FP: TCAAGACACCAACACATCGCA

TaqMan Probe: 5’ FAM-CAGGAGCCAGCCGTCGCACACCGT-TAMARA 3’

let-7g-5p：ugagguaguaguuuguacaguu

let-7g-PP: aaactactacctcaAGACACCAACACATCGCAATCAGGAGCCAGCCGTCGCACACCGTaactgtaca

let-7g-FP: TGAGGTAGTAGTTTGTACAGTTACG

let-7g-RP: TCAAGACACCAACACATCGCA

TaqMan Probe: 5’ FAM-CAGGAGCCAGCCGTCGCACACCGT-TAMARA 3’

let-7i-5p：ugagguaguaguuugugcuguu

let-7i-PP: aaactactacctcaAGACACCAACACATCGCAATCAGGAGCCAGCCGTCGCACACCGTaacagcaca

let-7i-FP: TGAGGTAGTAGTTTGTGCTGTTAC

let-7i-RP: TCAAGACACCAACACATCGCA

TaqMan Probe: 5’ FAM-CAGGAGCCAGCCGTCGCACACCGT-TAMARA 3’

let-7d-5p：agagguaguagguugcauaguu

let-7d-PP: aacctactacctctAGACACCAACACATCGCAATCAGGAGCCAGCCGTCGCACACCGTaactatgca

let-7d-FP: AGAGGTAGTAGGTTGCATAGTTAC

let-7d-RP: TCTAGACACCAACACATCGCA

TaqMan Probe: 5’ FAM-CAGGAGCCAGCCGTCGCACACCGT-TAMARA 3’

let-7e-5p：ugagguaggagguuguauaguu

let-7e-PP: aacctcctacctcaAGACACCAACACATCGCAATCAGGAGCCAGCCGTCGCACACCGTaactataca

let-7e-FP: TGAGGTAGGAGGTTGTATAGTTACG

let-7e-RP: TCAAGACACCAACACATCGCA

TaqMan Probe: 5’ FAM-CAGGAGCCAGCCGTCGCACACCGT-TAMARA 3’

miR-16-1-5p: uagcagcacguaaauauuggcg

miR-16-PP: tttacgtgctgctaAGACACCAACACATCGCAATCAGGAGCCAGCCGTCGCACACCGTcgccaatat

miR-16-FP: TAGCAGCACGTAAATATTGGCG

miR-16-RP: CTAAGACACCAACACATCGCA

Probe: 5’ FAM-CAGGAGCCAGCCGTCGCACACCGT-TAMARA 3’

miR-20a-5p: uaaagugcuuauagugcagguag

miR-20-PP: actataagcactttaAGACACCAACACATCGCAATCAGGAGCCAGCCGTCGCACACCGTctacctgca

miR-20-FP: TAAAGTGCTTATAGTGCAGGTAGA

miR-20-RP: TTAAGACACCAACACATCGCA

Probe: 5’ FAM-CAGGAGCCAGCCGTCGCACACCGT-TAMARA 3’

miR-21-5p: uagcuuaucagacugauguuga

miR-21-PP: agtctgataagctaAGACACCAACACATCGCAATCAGGAGCCAGCCGTCGCACACCGTtcaacatca

miR-21-FP: TAGCTTATCAGACTGATGTTGAAC

miR-21-RP: CTAAGACACCAACACATCGCA

Probe: 5’ FAM-CAGGAGCCAGCCGTCGCACACCGT-TAMARA 3’

miR-22-3p: aagcugccaguugaagaacugu

miR-22-PP: tcaactggcagcttAGACACCAACACATCGCAATCAGGAGCCAGCCGTCGCACACCGTacagttctt

miR-22-FP: AAGCTGCCAGTTGAAGAACTGT

miR-22-RP: CTTAGACACCAACACATCGCA

Probe: 5’ FAM-CAGGAGCCAGCCGTCGCACACCGT-TAMARA 3’

1. **Compare the performance of the singleplex and multiplex assays**

| MiRNA target | Multiplex RT-PCR | | |  | Singleplex RT-PCR | | |
| --- | --- | --- | --- | --- | --- | --- | --- |
|  | Liver | Heart | Lung |  | Liver | Heart | Lung |
| miR-16 | 24.3 | 21.8 | 23.7 |  | 23.8 | 21.0 | 24.6 |
| miR-20 | 25.7 | 24.2 | 24.2 |  | 26.2 | 24.8 | 24.2 |
| miR-21 | 24.8 | 26.6 | 21.9 |  | 23.7 | 26.9 | 22.2 |
| miR-22 | 28.5 | 26.0 | 29.7 |  | 28.7 | 24.8 | 29.8 |
| Let-7a | 27.2 | 24.6 | 24.8 |  | 26.3 | 23.2 | 23.6 |
| Let-7b | 27.8 | 27.9 | 25.6 |  | 27.1 | 27.2 | 23.9 |
| Let-7c | 25.2 | 22.9 | 23.4 |  | 25.3 | 23.9 | 22.6 |
| Let-7d | 29.8 | 27.2 | 29.1 |  | 27.9 | 26.5 | 29.2 |
| Let-7e | 34.4 | 32.5 | 30.1 |  | 33.6 | 31.0 | 29.1 |
